# Small protein mediates inhibition of ammonium transport in *Methanosarcina mazei*—an ancient mechanism?

**DOI:** 10.1128/spectrum.02811-23

**Published:** 2023-11-01

**Authors:** Tim Habenicht, Katrin Weidenbach, Adrian Velazquez-Campoy, Ruben M. Buey, Monica Balsera, Ruth A. Schmitz

**Affiliations:** 1 Institut für allgemeine Mikrobiologie, Christian-Albrechts-Universität zu Kiel, Kiel, Germany; 2 Institute of Biocomputation and Physics of Complex Systems (BIFI), Universidad de Zaragoza, Zaragoza, Spain; 3 Departamento de Bioquímica y Biología Molecular y Celular, Universidad de Zaragoza, Zaragoza, Spain; 4 Instituto de Investigaciones Sanitarias de Aragón (IIS Aragón), Zaragoza, Spain; 5 Centro de Investigación Biomédica en Red de Enfermedades Hepáticas y Digestivas (CIBEREHD), Madrid, Spain; 6 Departamento de Microbiología y Genética, Universidad de Salamanca, Salamanca, Spain; 7 Instituto de Recursos Naturales y Agrobiología de Salamanca, Spanish National Research Council (IRNASA-CSIC), Salamanca, Spain; The Pennsylvania State University, University Park, Pennsylvania, USA

**Keywords:** small protein, ammonium transport, protein regulation, pII protein, archaea, membrane proteins

## Abstract

In the past decade, small open reading frames (sORFs) coding for proteins less than 70 amino acids (aa) in length have moved into the focus of science. sORFs and the corresponding small proteins have been recently identified in all three domains of life. However, the majority of small proteins remain functionally uncharacterized. While several bacterial small proteins have already been described, the number of identified and functionally characterized small proteins in archaea is still limited. In this study, we have discovered that the small protein 36 (sP36), which consists of only 61 aa, plays a critical role in regulating nitrogen metabolism in *Methanosarcina mazei*. The absence of sP36 significantly delays the growth of *M. mazei* when transitioning from nitrogen limitation to nitrogen sufficiency, as compared to the wild type. Through our *in vivo* experiments, we have observed that during nitrogen limitation, sP36 is dispersed throughout the cytoplasm; however, upon shifting the cells to nitrogen sufficiency, it relocates to the cytoplasmic membrane. Furthermore, an *in vitro* biochemical analysis clearly showed that sP36 interacts with high affinity with the ammonium transporter AmtB_1_ present in the cytoplasmic membrane during nitrogen limitation as well as with the PII-like protein GlnK_1_. Moreover, the *in vivo* GlnK_1_ interaction with AmtB_1_ due to nitrogen upshifts requires the presence of sP36. Based on our findings, we propose that in response to an ammonium upshift, sP36 targets the ammonium transporter AmtB_1_ and inhibits its activity by mediating the interaction with GlnK_1_.

**IMPORTANCE:**

Small proteins containing fewer than 70 amino acids, which were previously disregarded due to computational prediction and biochemical detection challenges, have gained increased attention in the scientific community in recent years. However, the number of functionally characterized small proteins, especially in archaea, is still limited. Here, by using biochemical and genetic approaches, we demonstrate a crucial role of the small protein sP36 in the nitrogen metabolism of *M. mazei*, which modulates the ammonium transporter AmtB1 according to nitrogen availability. This modulation might represent an ancient archaeal mechanism of AmtB1 inhibition, in contrast to the well-studied uridylylation-dependent regulation in bacteria.

## INTRODUCTION

Small proteins are a significant part of the proteome in all organisms across the tree of life. The definition of this class of proteins is based on their small size rather than a functional criterion, which is typically defined by a cutoff ranging from 70 to 100 amino acids (1 – 5). Unlike peptides produced through posttranslational processing of larger proteins, the small proteins we refer to here are encoded by independent small open reading frames (sORFs). These sORF-encoded proteins, with a length of fewer than 70 amino acids (aa), have been historically overlooked and understudied due to bioinformatic biases inherent in conventional genome annotations and technical challenges associated with classical biochemical approaches, such as SDS-PAGE or mass spectrometry. Conventional gene annotations were primarily designed to identify larger proteins (6, 7), while proteomic tools relied on obtaining multiple peptides of one protein through tryptic digestion, which is often not feasible for small proteins (8). These challenges have impeded the annotation and characterization of small proteins in the past, creating a promising avenue for detailed mechanistic studies and functional analysis today, with high untapped potential.

Genome-wide transcriptomic, translatomic, and proteomic methods have been improved and developed in recent years to address and overcome the challenges of identifying the small proteome. The application of deep-sequencing technologies, as well as improvements and adaptation of ribosome profiling tools to bacteria and archaea, and optimized peptidome analyses by mass spectrometry allowed the identification of a constantly growing number of sORFs and the respective small proteins in bacteria and archaea (9 – 14). As a result, an increasing number of reports on small proteins encoded by sORFs are currently emerging and their physiological importance has been proven in numerous examples, by participation in various cellular functions such as cell division, transport, and enzymatic processes (3, 15, 16).

Since small proteins have come in the focus of science, it becomes more evident that a significant amount of proteins with less than 70 aa are associated with the cytoplasmic membrane. For *Escherichia coli*, a large portion of identified small proteins were shown to be localized at the membrane, where they might interact with larger proteins and protein complexes such as signal receptors or transporters (17 – 19). However, archaea exhibit fewer identified small proteins, and functional characterization is limited to a handful of examples, of which only one is part of a transporter (20 – 23).

*Methanosarcina mazei* strain Gö1 belongs to the order Methanosarcinales and is strictly anaerobic. This versatile group of methylotrophic archaea can utilize a variety of substrates, including methanol, methylamines, and acetate, in addition to CO_2_ and H_2_, as a source of both carbon and energy, with the ultimate product being methane, a greenhouse gas (24, 25). In the absence of another suitable nitrogen source, *M. mazei* is able to reduce and fix molecular nitrogen (26). This highly energy-consuming process of nitrogen fixation as well as the general nitrogen metabolism are strictly regulated on transcriptional, posttranscriptional, and posttranslational levels in response to nitrogen availability, which has been studied extensively in recent years (27 – 33). As known for bacteria, several key components of the nitrogen metabolism are only present and highly expressed under N-starvation, for example, glutamine synthetase, ammonium transporters, diazotrophs, and also nitrogenase (32, 34 – 37).

Under a sufficient ammonium concentration, nitrogen assimilation in cells occurs through the diffusion of ammonia across the cytoplasmic membrane and its subsequent incorporation into glutamate by glutamate dehydrogenase. However, under conditions of a significantly decreased external ammonium concentration, active transport of ammonium becomes necessary. This transport is facilitated by the trimeric ammonium transporter AmtB_1_, which is expressed exclusively under nitrogen (N) limitation in *M. mazei* (10, 38) but requires the expenditure of ATP (39, 40). Following transport, ammonium assimilation is facilitated by the glutamine synthetase/GOGAT (glutamine oxoglutarate aminotransferase) system (41). N starvation leads to elevated levels of 2-oxoglutarate (2-OG) within cells, which serves as an internal signal for N starvation (32).

AmtB_1_ is an ammonium transporter protein of the Amt/Mep/Rh protein family, of which, members can be found in eukaryotes, bacteria, as well as archaea. Through all domains of life, proteins of the Amt family show a highly conserved tertiary structure of 11 transmembrane helices with extracellular N- and cytoplasmic C- terminal domains (42, 43). In *Escherichia coli*, the ammonium transporter AmtB organizes as trimers with each subunit representing a hydrophobic pore for ammonia transport (44 – 46). The import of NH_4_^+^ is an energy-consuming process (39, 40). Consequently, the transporter is highly regulated depending on the nitrogen status of the cell to exclude energy dissipation. Based on the structure and complex formation analysis, Coutts et al. (46) showed that in *E. coli*, the ammonium transporter is inhibited by a PII-like protein (GlnK) upon a shift to nitrogen sufficiency. This regulation is based on GlnD-dependent deuridylylation of GlnK. The respective *glnK* gene is organized together with the *amtB* gene in an operon (*glnK/amtB*), which is only expressed under nitrogen starvation (47). This coupling of genes encoding an ammonium transporter and a PII-like protein has been identified in most bacteria as well as archaea, indicating a tight functional coexistence (48). However, GlnD, responsible for uridylylation and deuridylylation of GlnK, is not as ubiquitous. Those organisms with a *glnK/amtB* operon but without a *glnD* gene must rely on a different pathway of AmtB regulation.

A large number of potential sORFs were identified in *M. mazei* under N stress conditions through a genome-wide RNA sequencing (RNAseq) analysis (10). Among these, sORF36 encodes a 61 aa protein (sP36) that was confirmed by an LC-MS/MS analysis. Its transcription was shown to increase 2.5-fold under nitrogen limitation, as confirmed at the protein level. Additionally, sORF36 and sP36 are highly conserved on the DNA and protein level, across various archaeal species, suggesting a possible role of sP36 in nitrogen metabolism (31).

In this study, we characterize this additional newly discovered component in nitrogen regulation in *M. mazei*, the small protein sP36 (31). Through genetic and biochemical approaches, we show that sP36 is involved in the adaption to changing nitrogen conditions. Although sP36 does not contain any transmembrane helices, we provide several lines of evidence *in vitro* and *in vivo* that sP36 localizes at the cytoplasmic membrane in response to an ammonium upshift after a period of nitrogen limitation. Using different biochemical approaches, we demonstrated that the observed interaction with the cytoplasmic membrane is the result of the direct interaction between sP36 and the membrane-located ammonium transporter AmtB_1_. In a pull-down assay, purified His_6_-tagged AmtB_1_, incubated with a native *M. mazei* cell extract, is capable of specifically mediating the retention of chromosomally expressed sP36. Further biochemical analysis demonstrated a high-affinity interaction between sP36 and not only the ammonium transporter AmtB_1_ but also the regulatory PII-like protein GlnK_1_.

We propose a plausible model where sP36 acts as an adaptor protein that mediates the GlnK_1_–AmtB_1_ interaction to allow a rapid and reversible response to changes in nitrogen availability. This mechanism might represent a more ancient version of the AmtB inhibition by a PII-like protein before the GlnD-dependent uridylylation was developed.

## RESULTS

### sP36 plays a crucial role during nitrogen upshifts after a period of N limitation

**Fig 1:**
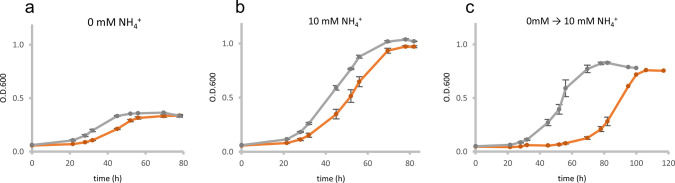
Growth analysis of *M. mazei* ∆sP36 in comparison to *M. mazei* wt: Growth of the *M. mazei* sP36 deletion mutant (∆sP36), (orange), and the wildtype, (gray), grown under different nitrogen availabilities with either 0 mM (**a**) or 10 mM (**b**) NH_4_^+^ in the medium or shifted from 0 mM to 10 mM (**c**). The respective NH_4_^+^ concentration is depicted. In each case, a 50 mL anaerobic minimal medium was inoculated with 1.6 × 10^8^ cells (**T_0_**). The standard deviation of the three biological replicates are shown.

To get insights into the functional role of sP36, a genetic approach was performed. A chromosomal deletion mutant of the respective sORF-encoding sP36 was constructed, replacing the *sORF36* gene with the puromycin-resistance cassette (*pac* cassette) using an allelic replacement approach (see Materials and Methods). The generated mutant strain (*M. mazei* ΔsP36) was verified by Southern blot analysis (Fig. S1). Its growth behavior under different N availabilities was evaluated and compared to the wild type strain (*M. mazei* 3A (wt, selected for improved growth on solid media); Fig. 1). When growing on a minimal medium with ammonium as a sufficient N source (10 mM) or under N limitation (0 mM), no growth phenotype was detectable in the absence of sP36 except that the lag phase was slightly prolonged but reaching identical doubling times. However, when cells were grown under N limitation until early exponential phase (turbidity at 600 nm (T_600_) = 0.15) and then transferred into fresh ammonium-sufficient media (1.6 × 10^8^ cells in 50 mL media), the cultures of *M. mazei* ΔsP36 showed a significantly prolonged phase of adaptation (38 h lag phase) in the ammonium-sufficient medium, before again entering an exponential growth phase reaching the same doubling time as the wild type. In contrast, the wild type (wt) immediately entered exponential growth after the shift to ammonium sufficiency. These findings strongly indicate the crucial role of sP36 under nitrogen upshift conditions.

**Fig 2:**
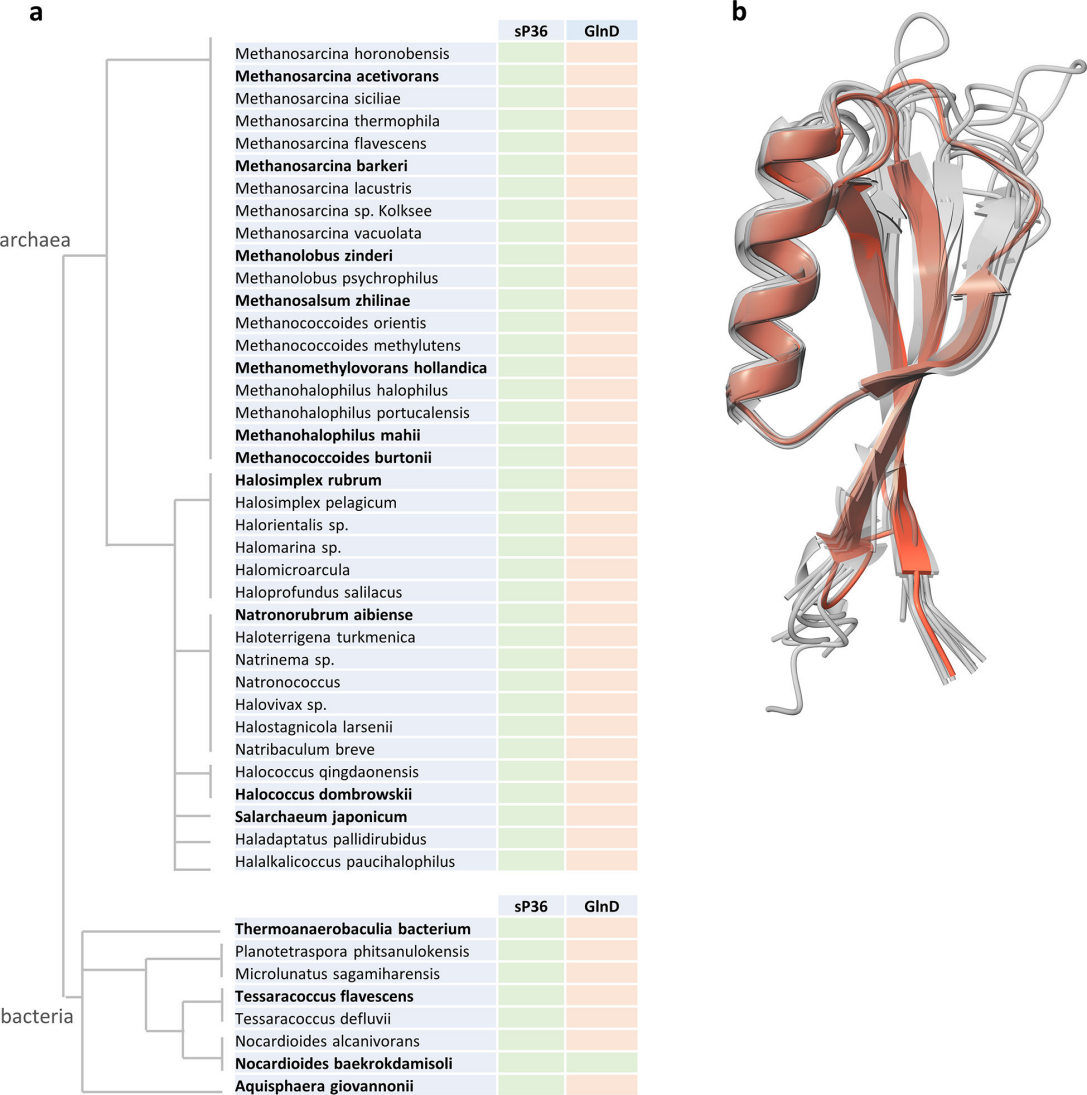
Conservation of sP36. (a) sP36 is conserved in archaea and bacteria. sP36 homologs were found in archaeal and bacterial species. Species which do not encode GlnD are marked in the GlnD column in orange, and those which encode GlnD are marked in green. Taxonomic grouping is based on the Genome Taxonomy Database (51). (b) sP36 structural conservation based on alphaFold2 prediction. sP36 from *M. mazei* is given in yellow. Organisms of which homologs were used for structure predictions are highlighted in bold.

We performed a basic local alignment search tool analysis with the *M. mazei* sP36 amino acid sequence (49). Homologs of sP36 were found in a high number of methanogenic and halophilic archaea in seven archaeal families in the two classes of Methanosarcinia and Halobacteria inside the phylum of halobacteriota. Even in the six bacterial families (streptosporangiaceae, propionibacteriaceae, nocardioidaceae, isophaeraceae and one thermoanaerobaculia family), homologs of sP36 were found (see Fig. 2a). Interestingly, those organisms encoding sP36 do not encode a homolog of the uridylyltransferase GlnD, which is required for the uridylyl-dependent AmtB regulation by GlnK, as described in the Introduction. The different homologs of sP36 show 37% to 95% identity on an amino acid level but very high structural conservation based on alphaFold2 (50) predictions (see Fig. 2b).

### sP36 localizes at the cytoplasmic membrane in response to a shift from N limitation to N sufficiency

**Fig 3:**
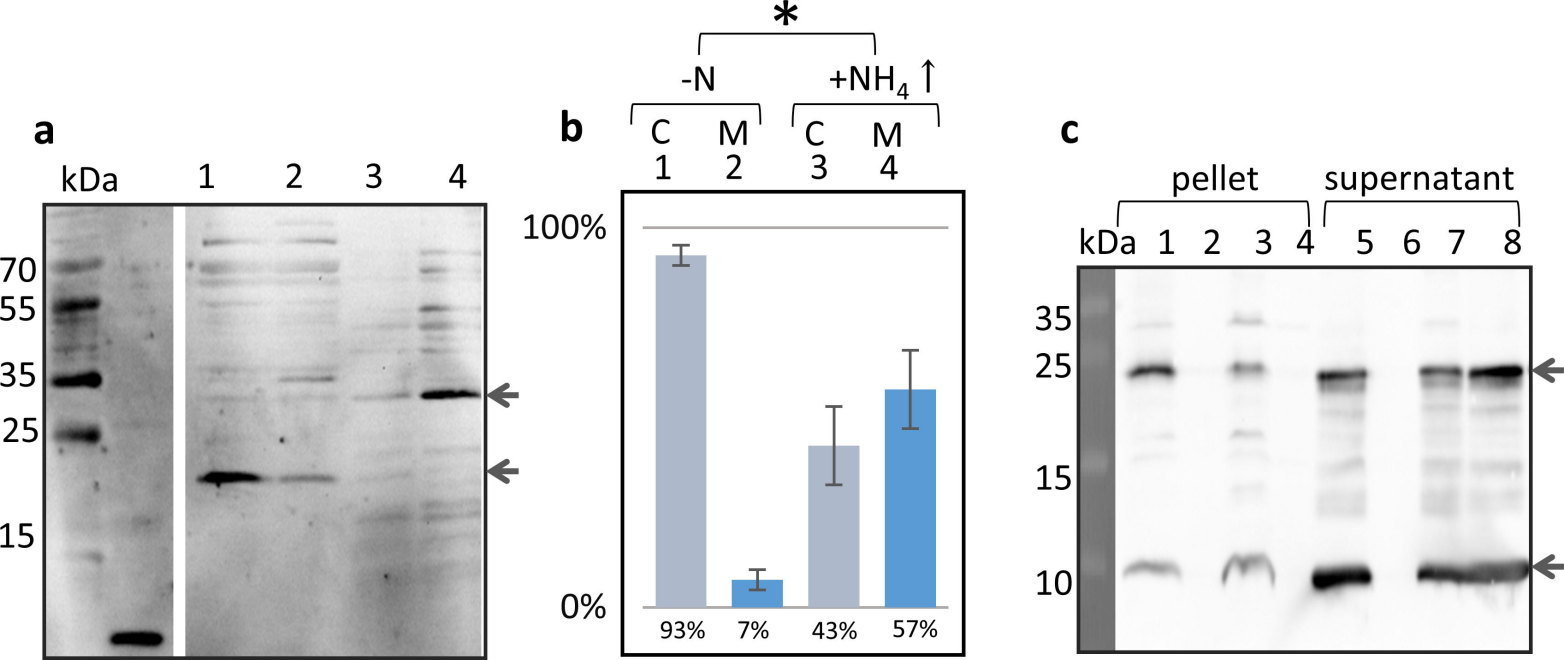
Interaction of sP36 with the cytoplasmic membrane of *M. mazei* under different N conditions. A + B: *M. mazei* cultures were grown under N limitation (0 mM NH_4_^+^ N_2_, (-N). When T_600_ of 0.2 was reached, 50% of the cultures were shifted to N sufficiency (10 mM NH_4_^+^ final concentration, NH_4_￪). (**a)** Membrane and the cytoplasmic fractions were analyzed by western blot with a polyclonal antibody raised against sP36. (1) -N cytoplasmic fraction; (2) NH_4_￪ cytoplasmic fraction; (3) -N membrane fraction; and (4) NH_4_￪ membrane fraction. The major sP36 signals are indicated by arrows. Depicted is one exemplary western blot out of three biological replicates. (b) Relative quantification of the dominant bands of sP36 subcellular fractions from *M. mazei.* (1) -N cytoplasmic fraction; (2) -N membrane fraction; (3) NH_4_￪ cytoplasmic fraction; (4) NH_4_￪ membrane fraction; the distribution of the sP36 subcellular localization was calculated based on three biological replicates. The amount of sP36 in the cytoplasm and the membrane fraction of one culture was set to 100%. Significance was tested using the two-tailed *t*-test. *P = 0.014; df = 4. (c) *In vitro* interaction of sP36 with *M. mazei* ∆sP36 membrane fractions: untagged sP36 (100 µg derived from His_6_-SUMO-sP36) was incubated together with the membrane fraction of *M. mazei* ∆sP36 subcellular fractionation. Lanes 1–4: pellet of 210,000 *g* centrifugation; lane 1: sP36 incubated with the –N membrane fraction of *M. mazei* ∆sP36; lane 2: -N membrane fraction of *M. mazei* ∆sP36 without sP36 (control); lane 3: sP36 incubated with the membrane fraction of *M. mazei* ∆sP36 grown under N sufficiency (+N); lane 4: pellet of sP36 without membrane fraction (control); lanes 5–8: supernatant of 210,000 *g* centrifugation; lane 5: sP36 incubated with the -N membrane fraction of *M. mazei* ∆sP36; lane 6: -N membrane fraction of *M. mazei* ∆sP36 without sP36 (control); lane 7: sP36 incubated with the membrane fraction of *M. mazei* ∆sP36 grown under N sufficiency (10 mM); lane 8: sP36 without membrane fraction (control). The major sP36 signals are indicated by arrows. Depicted is one exemplary western blot out of three biological replicates.

The cellular localization of sP36 under N-limited growth conditions (-N) and after an ammonium upshift was evaluated by subcellular fractionation of the cell extract and subsequent western blot analysis using peptide antibodies directed against sP36. One liter of *M. mazei* cultures were grown under -N. When reaching mid-exponential growth phase (T_600_ = 0.2), 50% of the cultures were shifted to N sufficiency by supplementing with 10 mM ammonium (final concentration). The remaining 50% were kept N-limited. After further incubation for 30 min, subcellular fractionation was conducted as described in Materials and Methods, followed by a western blot analysis of the respective cytoplasmic and membrane fractions using specific peptide antibodies against sP36. Overall, three independent biological replicates were analyzed, each with three technical replicates. Under –N, sP36 was predominantly dispersed in the cytoplasm (93 ±3%). However, upon ammonium upshift, most of sP36 relocated into the membrane fraction (57 ±10%; Fig. 3b). In addition to the shift in localization, there is an apparent change in the molecular weight of the sP36 signal. This change is most likely the result of sP36 oligomerization, which is induced by the shift to increased ammonium concentration *in vivo* or the interaction with a protein in the cytoplasmic membrane.

The observed interaction between sP36 and the cytoplasmic membrane was further verified in an *in vitro* assay using purified tag-less sP36 and cytoplasmic membrane fractions. Membrane fractions from the mutant *M. mazei* strain ΔsP36 grown under -N as well as after an ammonium upshift (+NH_4_^+^↑) were generated by ultracentrifugation as described in Materials and Methods. The heterologously expressed, purified tag-less sP36 (100 µg) was incubated in the presence of the *M. mazei* ΔsP36 membrane fractions (5 mg) for 15 min at RT (room temperature) followed by ultracentrifugation at 210,000 *g* and 4°C for 1 h. The respective pellet and supernatant were evaluated for the presence of sP36 by western blot analysis using peptide antibodies against sP36 (see Fig. 3c). In the control sample of the *M. mazei* ΔsP36 membrane (-N) without prior incubation with sP36, no signal was detected, neither in the supernatant (lane 6) nor in the membrane fraction (pellet, lane 2), confirming the absence of sP36 in the *M. mazei* ΔsP36 mutant strain as well as the specificity of the antibody. In the absence of membrane fractions, purified sP36 was exclusively present in the supernatant after ultracentrifugation (lane 8 vs lane 4). However, when incubated in the presence of the cytoplasmic membrane fraction of cells grown under nitrogen limitation, approximately 50% of sP36 was detected in the membrane fraction (lane 1, pellet), strongly arguing for a recruiting of the soluble hydrophilic sP36 to the membrane. Even when cells were grown under nitrogen sufficiency, part of the sP36 was detectable in the membrane fraction after centrifugation (lane 3).

### sP36 interacts with ammonium transport proteins

**Fig 4:**
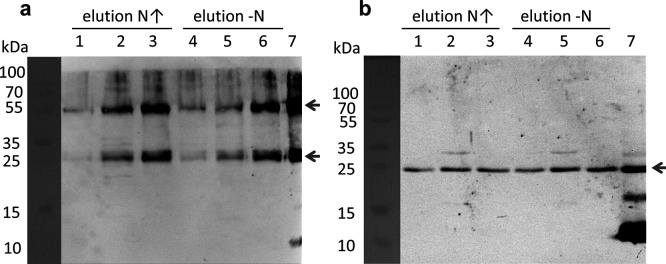
Pulldown with AmtB_1_-His_6_ as bait against *M. mazei* cell extract grown under N-limited (–N) and shifted to nitrogen-sufficient (N↑) condition. (**a)** Western blot using the anti-His-tag antibody shows AmtB_1_ in the elution fractions of the N↑ and –N pulldowns. Indicated by a black arrow are the monomeric AmtB_1_ at approximately 30 kDa and the dimeric AmtB_1_ at 55 kDa. (**b)** Western blot with the specific sP36 peptide antibodies shows coelution of chromosomal-expressed native sP36 from the *M. mazei* cell extract with AmtB_1_-His_6_. 1–3: elutions of the pulldown using cell extract of cells grown under N upshift, 4–6: elutions of the pulldown using cell extract of cells grown under N-limited condition, 7: positive controls, purified AmtB_1_-His_6_ (**a**) and purified sP36 (**b**), respectively.

**Fig 5:**
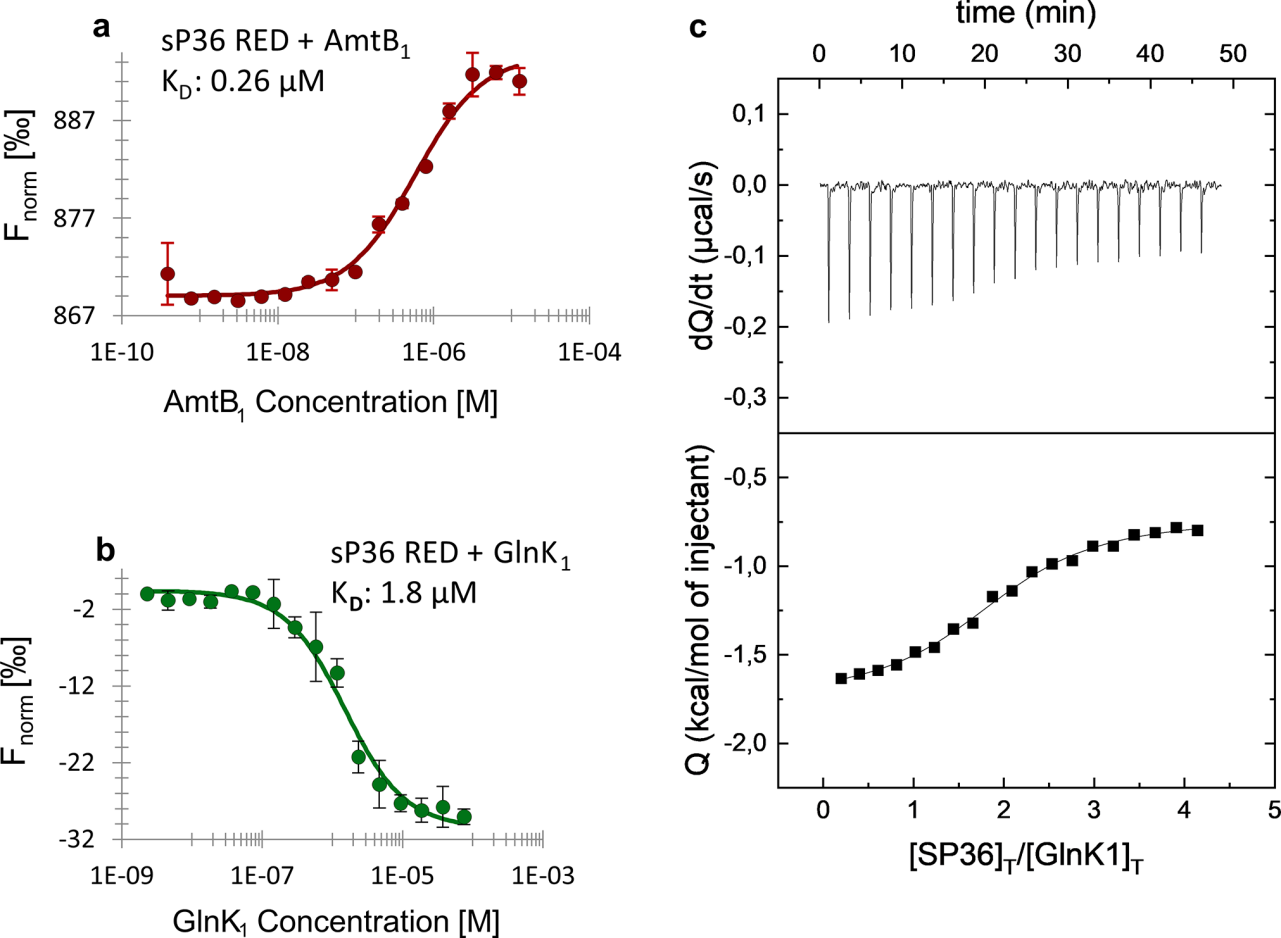
sP36 interaction studies using MST and isothermal titration calorimetry (ITC). (a) Interaction studies between sP36 and AmtB_1_-His_6_ using MST. RED-labeled untagged sP36 was used at 100 nM and AmtB_1_-His_6_ at different concentrations ranging from 12.5 µM to 0.38 nM, calculated based on the monomeric molecular mass. Based on three biological replicates, the *K*_D_ was estimated to be 0.26 µM (± 0.07 µM). (b) Interaction studies between sP36 and GlnK_1_ using MST. RED-labeled untagged sP36 at 100 nM and His_6_-GlnK_1_ at 16 different concentrations ranging from 7.5 µM to 0.23 nM were used for MST analysis, resulting in a dissociation constant *K*_D_ of 1.8 µM (± 1.1 µM). In both cases (**a and b**), exemplarily one of the three biological replicates is depicted. (c) Interaction studies between sP36 and GlnK_1_ using ITC. 20 µM His_6_-GlnK_1_ was titrated with 300 µM at 25°C. Control experiments were performed by injecting sP36 into buffer. The dissociation constant *K*_D_ was evaluated to be 5.4 µM. The sP36–GlnK_1_ interaction has a stoichiometry of 2:1 as described in Materials and Methods.

The subcellular localization experiments as shown above strongly suggest localization of sP36 to the cytoplasmatic membrane upon an ammonium upshift. One potential interacting partner of sP36 in the cytoplasmic membrane is the ammonium transporter AmtB_1_ because it is only expressed under N limitation (38) and is a key component under N limitation for transporting residual ammonium into the cell, which critically requires to be inhibited upon an upshift. To test this hypothesis, a pull-down experiment with purified C-terminal His_6_-tag AmtB_1_ was performed using crude cell extracts of *M. mazei* either grown under N limitation or under N limitation but shifted to ammonium sufficiency (0 → 10 mM) in an exponential growth phase for 30 min. After incubating AmtB_1_-His_6_ with 25 mg of the total cell extract, a Ni-NTA affinity chromatography was performed and the elution fractions were analyzed by SDS-PAGE and western blot using the anti-His tag and anti-sP36 antibodies. The results clearly show that exogenous AmtB_1_-His_6_ and native sP36 co-elute independent of the nitrogen conditions in which the cells were grown (Fig. 4). These findings strongly suggest that AmtB_1_ forms a complex with sP36. The direct interaction between sP36 and AmtB_1_ was further confirmed and evaluated by microscale thermophoresis (MST) using recombinant AmtB_1_-His_6_ and untagged sP36 (RED) resulting in an estimated dissociation constant of *K*_D_ = 0.26 ± 0.07 µM (Fig. 5a).

As stated in the Introduction, in bacteria, the PII-like protein GlnK is known to interact with the ammonium transporter AmtB to modulate the transport activity of AmtB in response to an ammonium upshift (52). Therefore, we next aimed to evaluate the interaction between sP36 and GlnK_1_ by using recombinant N-terminal his-tagged GlnK_1_ (His_6_-GlnK_1_) and sP36 (RED) proteins by MST, which yielded a dissociation constant of 1.8 ± 1.1 µM (Fig. 5b).

The interaction between sP36 and GlnK_1_ was further verified by isothermal titration calorimetry (ITC, Fig. 5c). The ITC analysis clearly verified the interaction and demonstrated that sP36 forms a complex with GlnK_1_ with a dissociation constant *K*_D_ of 5 µM in a 2:1 stoichiometry, that is, each monomer of GlnK_1_ binds two sP36 monomers.

**Fig 6:**
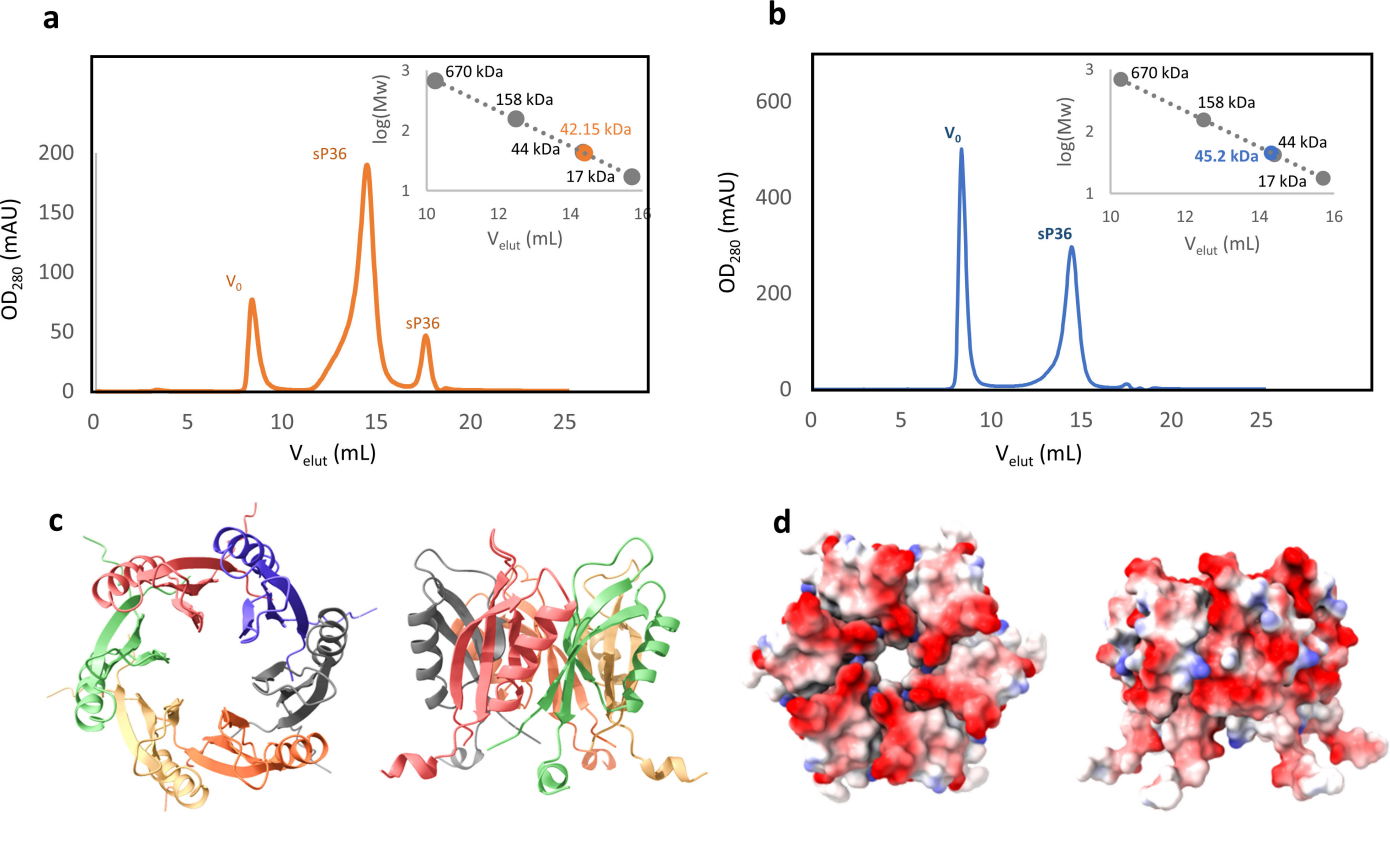
Oligomerization studies of purified sP36. (a) Untagged sP36 from expression in *E. coli*. Proteins and protein complexes were separated by SEC. The fractions of the main elution peak (retention volume of 14 mL to 15 mL) correspond to the molecular mass of the sP36 hexamer (42.6 kDa). Orange, sP36-His_6_; gray, size-exclusion standard.** (b)** His_6_-sP36 from expression in *M. mazei*. Proteins and protein complexes were separated in a SEC. The fractions of the main elution peak (retention volume of 14 mL to 15 mL) correspond to the molecular mass of the His_6_-sP36 hexamer (47.8 kDa). Blue, sP36-His_6_; gray, size-exclusion standard. (c and d) Structure prediction of a sP36 hexamer (50, 53). (**c)** Secondary structural elements of the sP36 hexamer, where each protomer is in a different color. (**d)** Electrostatic surface of the sP36 hexamer. The calculation was performed with the APBS plug-in implemented in PyMOL (Schrödinger Inc., 2015, The PyMOL Molecular Graphics System. Version 2.0 Schrödinger LLC). Color oscillates from −2.0 (red) to +2.0 (blue) KbT/ec.

Given that GlnK_1_ forms trimers in solution (27), a 2:1 stoichiometry aligns perfectly with the oligomeric state of sP36, which has been determined to form stable hexamers in solution by analytical size-exclusion chromatography (SEC) experiments. SEC was performed with sP36 from heterologous expression in *E. coli* as well as with His_6_-sP36 purified from *M. mazei* (Fig. 6a and b). Using AlphaFold2 for computational modeling, we generated a structural model of hexameric sP36 (Fig. 6c), whose statistical parameters pLDDT and PAE (Fig. S2) demonstrate that the residues are correctly positioned in their local environment, and the monomers are accurately positioned relative to each other (50). The hexameric configuration of sP36 adopts a truncated cone shape, with the negative charged surface at the narrower side (Fig. 6d).

**Fig 7:**
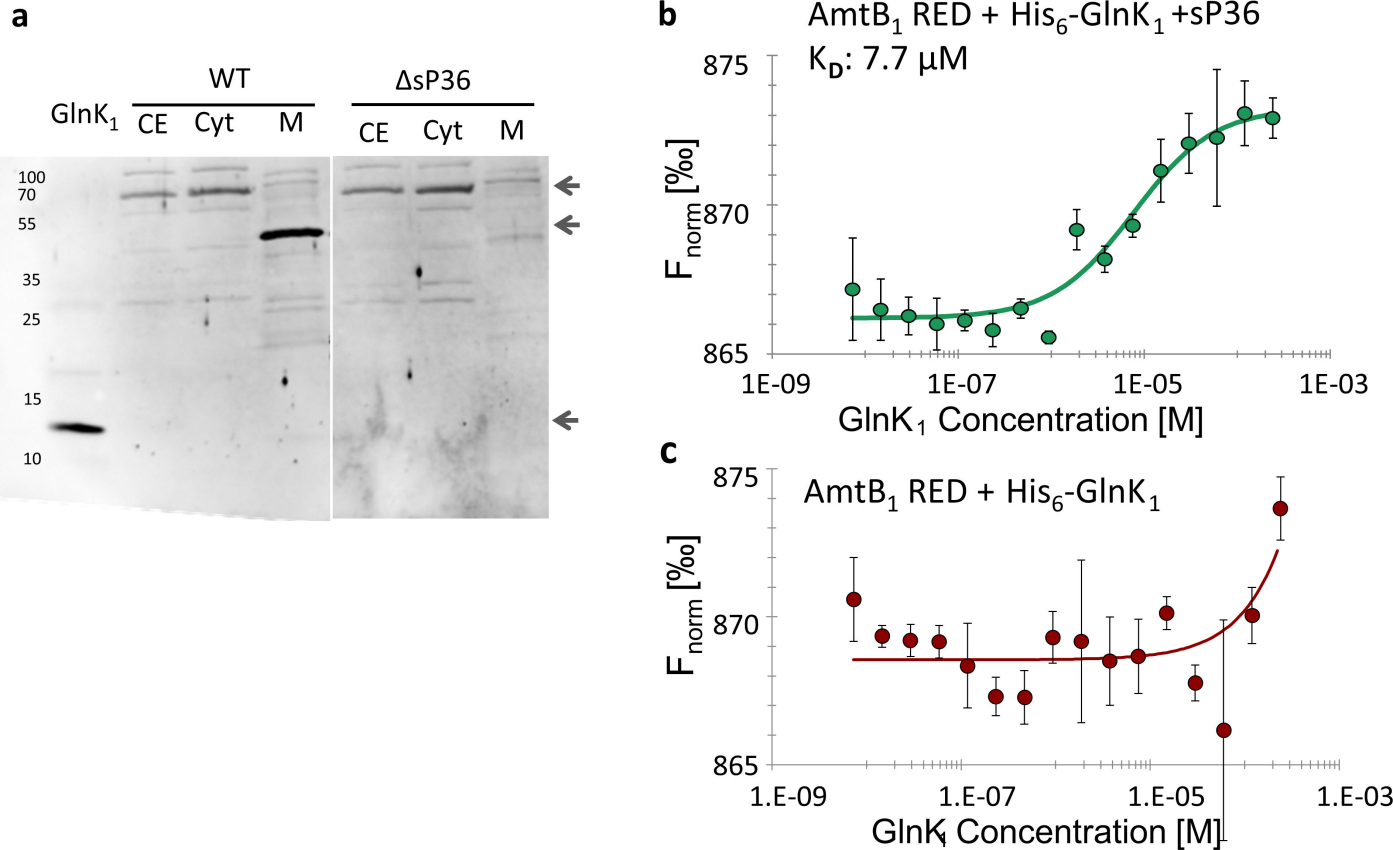
AmtB_1_–GlnK_1_ interaction is dependent on sP36. (a) Localization of GlnK_1_ in *M. mazei* ΔsP36. *M. mazei* wt and ΔsP36 cultures (50 mL) were grown under N limitation until reaching the exponential growth phase and then shifted to N sufficiency (10 mM). Subcellular fractions were generated. The cell extract (CE) and cytoplasmic (Cyt) and membrane (M) fractions were evaluated for GlnK_1_ presence by a western blot analysis using polyclonal antibodies against GlnK_1_. Cyt and M fractions from *M. mazei* wt(DSM3647) and ΔsP36 were analyzed and compared to purified His_6_-GlnK_1_. The major GlnK_1_ bands are indicated by arrows. Depicted is one exemplary western blot out of three biological replicates. (b and c) Interaction studies between AmtB_1_-His_6_ and GlnK_1_-His_6_ using MST. RED-labeled His_6_-tagged AmtB_1_ was used at 20 nM and GlnK_1_-His_6_ at different concentrations ranging from 480 µM to 14.6 nM, calculated based on the monomeric molecular mass. (b) In the presence of sP36 (480 µM), the *K*_D_ was estimated to be 7.7 µM (± 2.5 µM) based on three biological replicates. (c) In the absence of sP36, no interaction could be detected.

MST and ITC independently showed an interaction between sP36 and GlnK_1_ with a high affinity (*K*_D_ in the low µM range). Thus, we next aimed at evaluating the impact of sP36 on the subcellular localization of GlnK_1_. *M. mazei* wt and ΔsP36 strains were grown under N limitation followed by an ammonium upshift for 30 min, and the cultures were harvested in the mid-exponential growth phase. After subcellular fractionation, the presence of GlnK_1_ was detected in the cytoplasmic and membrane fractions by western blot with specific antibodies. In the wt strain, GlnK_1_ was predominantly present in the membrane fraction, while only a minor fraction was detected in the cytoplasm (Fig. 7). However, in ΔsP36, GlnK_1_ is no longer detected in the membrane fraction, but mostly in the cytoplasm. In both subcellular fractions, GlnK_1_ was detected at a different molecular weight, which corresponds to GlnK_1_ in a stable complex with either soluble or membrane proteins. Remarkably, the total amount of GlnK_1_ in the absence of sP36 appears to be decreased (Fig. 7a).

Since sP36 seems to be crucial for sequestration of GlnK_1_ to the membrane, the interaction of AmtB_1_ and GlnK_1_ in dependence of sP36 was studied *in vitro*. In MST, AmtB1 (RED) only interacts with His_6_-GlnK_1_ if sP36 is present in the reaction buffer. If no sP36 was added to the reaction, no difference in MST traces was observed and no interaction could be evaluated. However, in the presence of sP36, AmtB_1_ interacts with GlnK_1_ *in vitro* and a *K*_D_ of 7.7 µM was calculated (see Fig. 7b and c).

## DISCUSSION

Based on the aforementioned results, we propose that in *M. mazei* both GlnK_1_ and sP36 are required for the complete inhibition of AmtB_1_, the ammonium transporter, in response to an upshift in ammonium concentration after a period of N limitation in *M. mazei*. The essential role of sP36 in AmtB_1_ regulation during an N-upshift is further corroborated by our genetic analysis. The chromosomal mutant strain (*M. mazei* ∆sP36) displays a significantly prolonged lag phase when shifted from N limitation to ammonium sufficiency (10 mM NH_4_^+^) compared to the wild type strain. This strongly indicates that the ammonium transporter in the absence of sP36 retains significant activity, leading to an unnecessary cycle of active AmtB_1_-mediated import together with passive diffusion, resulting in excessive energy consumption. This might explain the significantly prolonged lag phase observed in the deletion mutant strain after a shift to N sufficiency.

The mechanism of AmtB regulation by the PII protein GlnK is well characterized in *E. coli*. Here*,* the cellular nitrogen status is perceived by GlnD, which transduces the signal to GlnK through a covalent modification. Under N limitation, GlnK is uridylylated at the Y51 residue in the T-loop by GlnD. In response to increased ammonium concentrations, GlnK is rapidly deuridylylated by GlnD and the demodified GlnK subsequently interacts with AmtB and inhibits its activity (46, 52). The trimeric GlnK binds AmtB with the T-loop of each monomer, physically blocking the hydrophobic pore of the AmtB trimer and the cytoplasmic pore exit (54). This regulatory mechanism appears to be highly conserved and has been also shown for *Rhodospirillum rubrum* (55), and the archaea *Haloferax mediterranei* (56, 57) and *Archaeoglobus fulgidus* (58). The *A. fulgidus* regulation was mainly proposed based on the structural studies of purified AmtB and by using a docking model for the interaction with the PII-like protein. Interestingly, the conserved T-loop of the *A. fulgidus* PII-like protein lacks the Y51 residue, which is the residue modified by uridylylation in *E. coli* (58).

In *M. mazei,* two copies of the *glnK*/*amtB* operon are present. While the *glnK_1_*/*amtB_1_* operon is highly regulated in response to N availability by NrpR, the *amtB_2_*/*glnK_2_* operon is not expressed under N limitation and has been proposed to be a potential backup system (38, 59). Although the T-loop of GlnK_1_ contains the conserved tyrosine residue (Y51), no posttranslational modification of GlnK_1_ could be observed in response to an ammonium upshift in *in vivo* and *in vitro* experiments (27). Moreover, no GlnD homolog is encoded in *M. mazei* (60) or in any archaeal genome. Consequently, in archaea like *M. mazei*, a potential GlnK_1_ regulation of AmtB_1_ requires a different mode of signal perception of changing nitrogen conditions.

**Fig 8:**
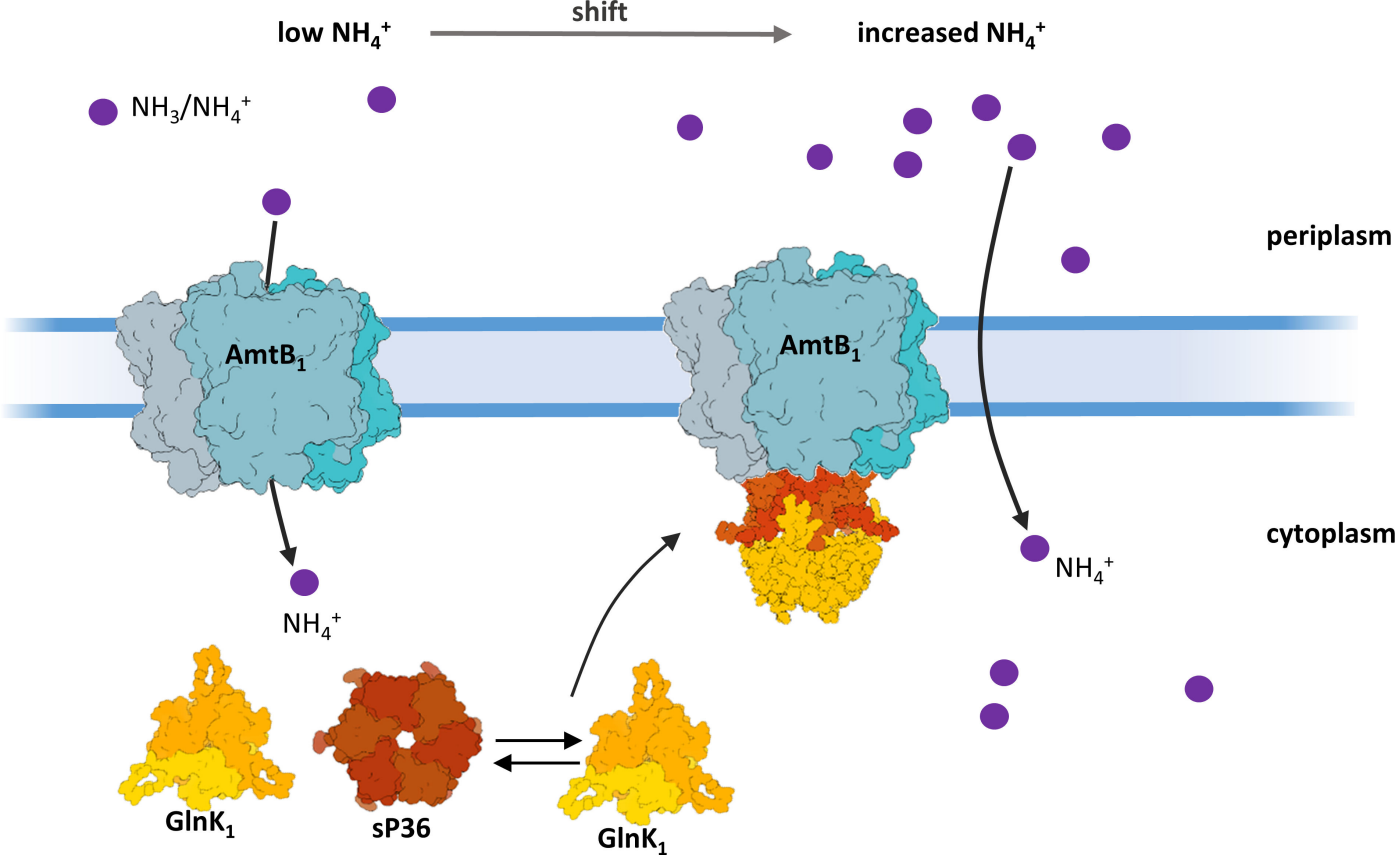
Hypothetical model of AmtB_1_ regulation in *M. mazei*. The AmtB_1_ trimer is actively importing ammonium under a low N concentration. However, after an upshift to ammonium sufficiency in the surrounding medium, sP36 is proposed to mediate the formation of a GlnK_1_-AmtB_1_ complex, allowing the GlnK_1_ trimer to block the AmtB_1_ activity to completeness. The displayed structures of GlnK1 and sP36 were generated using AlphaFold2 (50, 53) (Fig. S2). The structure of the ternary complex is a hypothetical model and requires further investigation.

Based on our results, we propose a hypothetical model for the regulation of AmtB_1_ by sP36 in response to an increase in N availability, which is summarized in Fig. 8. In the absence of combined nitrogen or when the ammonium concentration is very low, most of the sP36 is located in the cytoplasm, while AmtB_1_ actively transports the remaining NH_4_^+^ into the cell. When the external ammonium concentration increases (N-upshift), NH_3_ diffusion provides the cell with sufficient ammonium. Thus, the energy-consuming NH_4_^+^ transport by AmtB1 is inhibited through a direct protein–protein interaction with the GlnK_1_ trimer. This complex formation between trimeric AmtB_1_ and trimeric GlnK_1_, however, is crucially dependent on hexameric sP36, which binds both proteins with high affinity. Consequently, sP36 favors the AmtB_1_–GlnK_1_ interaction in response to an ammonium upshift after a period of N limitation. The mechanism which triggers sP36 to interact with AmtB1 after ammonium upshift is still uncertain. However, most likely, oligomerization of sP36 is shifted toward a hexamer after an increase of ammonium concentration. This change is possibly based on sensing the concentration of 2-oxoglutarate and/or glutamine, which reflects the cell intern nitrogen availability. The nitrogen signal, the structure of the binary and ternary complexes, and the hierarchy of the interactions require further investigation and are currently being studied in our laboratory. We also note that the symmetry and the negatively charged surface on the sP36 hexamer (Fig. 6d) are well-suited to interact with the positively charged intracellular side of AmtB_1_, as reported in the literature.

Finally, sP36 shows conservation in a broad range of methanogenic and halophilic archaea and is even present in the genome of some bacteria. It is worth noting that conservation of sP36 is coupled to an absence of the urydylyltransferase encoding *glnD*. Only a few bacteria also have a sP36 in addition to *glnD*. This might be due to horizontal gene transfer of the sP36 gene because those organisms are reported to grow in an highly diverse environment like animal intestinal tracts, also containing methanoarchaea. Consequently, the GlnD-dependent and the sP36-mediated pathways exhibit two different modes of AmtB regulation in separate groups of organisms. Due to the evolutionary placement of halophilic and methanogenic archaea, we speculate that sP36-mediated inhibition of AmtB_1_ represents the archaeal, more ancient mechanism for responding to changes in N availability, predating the AmtB inhibition by a PII-like protein, before the GlnD-dependent pathway evolved in bacteria.

## MATERIALS AND METHODS

### Construction of plasmids

#### sP36 genomic deletion

The flanking regions ~ 1,000 bp upstream and ~1,000 bp downstream of the sORF36 were amplified from genomic *M. mazei* DNA by using primers (Eurofins, Ebersberg, Germany) listed in Table S2. A puromycin resistance (Pur^R^)-mediating *pac-*cassette was restricted from pRS207. The 1,000 bp downstream fragment was restricted by enzymes *EcoRI* and *BamHI* and inserted into the multiple cloning site (MCS) of the vector pMCL210, resulting in pRS1305. The 1,000 bp upstream fragment was cleaved using *EcoRI* and *KpnI* and subsequently ligated in pRS1305. The resulting plasmid was designated pRS1307. The *pac*-cassette was ligated into the *EcoRI* site of pRS1307 and the resulting plasmid was named pRS1308. pRS1308 was linearized using *ScaI* and transformed into *M. mazei* wt (3A) cells by liposome-mediated transformation. Insertion in the chromosome occurred through double homologous recombination by selection for puromycin resistance^57^. The success of the allelic marker exchange of single-mutant colonies was verified in puromycin-containing media and further analyzed via Southern blot analysis with specific probes directed against the *sP36* gene and *pac*-cassette (Fig. S1).

#### sP36 overexpression

For cloning *MMsORF36* into pETSUMO, pET28a, and pWM321, a construct including the *pmcrB* promotor and (His)_6_-*MMsORF36* fusion was synthesized (Eurofins Genomics, Ebersberg, Germany). The plasmid was named pRS1214. *MMsORF36* was cloned into pETSUMO using pRS1214 as a template, primer pair sORF36_3for/sORF36_3rev, and the Champion pET SUMO expression system (Thermo Fisher Scientific, Waltham, USA) according to the manufacturer’s instructions, yielding plasmid pRS1240 and strain *E. coli* BL21 K4099. The cloning of *MMsORF36* into pET28a was done via an intermediate. First, *MMsORF36* was amplified with the primer pair sORF36_forNdeI/sORF36_3revNdeI (Table S2) using the template pRS1214 and subsequently ligated via TA cloning into the pCRII vector (Thermo Fisher Scientific, Waltham, USA) according to the manufacturer’s instructions. The resulting construct was designated pRS1223 in *E. coli* DH5α K4071. In the second step, the insert was excised from pRS1223 using the *NdeI* site and ligated into the *NdeI* site of pET28a. The resulting plasmid was designated pRS1225 and transformed in *E. coli* BL21 pRIL yielding the strain K4092. Upon cloning of *MMsORF36* in pWM321, the *pmcrB* promotor-(His)_6_-*MMsORF36* fusion was isolated using *SacI* and *KpnI* sites from pRS1214 and ligated into the corresponding sites of pWM321, resulting in plasmid pRS1227. pRS1227 was transformed into *M. mazei* wt (3A) as described before (53).

#### His_5_-SUMO-TEV-sP36 construct

A TEV (tobacco etch virus protease) cleavage site was inserted between the Sumo tag and sORF36 in the His_5_-SUMO-sP36 overexpression construct (pRS1240). Therefore, site-directed mutagenesis was conducted using primers SP36_TEV_rv and SP36_TEV_fw (Table S2).

### Growth of *M. mazei*

*M. mazei* was cultivated in sealed bottles of an anaerobic minimal medium with a gaseous phase consisting of N_2_ and CO_2_ (vol/vol, 80/20) (26, 60). The medium was supplemented with 150 mM methanol as carbon source, and in case of cultures growing in nitrogen sufficiency, additionally 10 mM ammonium chloride was used. Cells were cultivated until an optical turbidity of 0.5–0.6 at 600 nm (T_600_ = 0.5–0.6). –N Cells were grown until T_600_ = 0.2–0.3.

*M. mazei* cells were harvested by centrifugation at 4,000 *g* at 4°C for 30 min. The cells were resuspended in 2 mL of 50 mM Tris buffer (pH 7.6) and lysed by using a dismembrator (Sartorius, Göttingen, Germany) at 1,600 rpm for 3 min. The whole cell extract was centrifuged for 30 min at 13,000 *g* and 4°C to get rid of cell debris and the remaining unlysed cells.

### Subcellular fractionation

For subcellular fractionation of *M. mazei*, the cultures were grown anaerobically as described. Cells were harvested by centrifugation t 6,000 *g* at 4°C for 30 min. The cells were resuspended in 10 mL of Tris buffer (50 mM, pH 7.6) and afterward lysed by using a dismembrator (Sartorius, Göttingen, Germany) at 1,600 rpm for 3 min. The lysate was centrifuged for 30 min at 7,500 *g* and 4°C. To separate the membrane and cytoplasmic fractions, the supernatant was further centrifuged at 210,000 *g* for 1 h at 4°C.

### Purification of expressed proteins

#### His_6_-sP36, His_6_-GlnK1, and His_6_-SUMO-sP36

*E. coli* Bl21 (DE3)/pRIL cultures were grown in an LB (Luria-Bertani) medium at 37°C under continuous shaking. At T_600_ = 0.6, the protein expression was induced by adding 100 µM IPTG (isopropyl-β-thiogalactoside) (final concentration) and the cultures were incubated for 2 hours. Cells were harvested by centrifugation at 4,000 *g* at 4°C for 20 min, suspended in 6 mL of phosphate buffer A (50 mM phosphate, 300 mM NaCl, pH 8.0) and lysed by passing through a French pressure cell two times with 80 N(mm^2^)^−1^. Afterward. the extract was centrifuged for 30 min at 13,000 *g* and 4°C to get rid of cell debris and the remaining unlysed cells. For protein purification from *M. mazei*, cultures were grown and lysed as described above. His-tagged proteins were purified by affinity chromatography on Ni-NTA agarose (Qiagen, Hilden, Germany) gravity flow columns with 1 mL of bed volume. Proteins were eluted in steps with 100 mM, 250 mM, and 500 mM imidazole.

#### SUMO cleavage

For cleavage of the SUMO-(His)_6_-tag, 200 µL of SUMO-protease (Thermo Fisher Scientific, Waltham, MA, USA) per 1 mg of tagged protein was used. The reaction mixture was incubated for 1 h at 30°C. The cleaved sP36 protein was afterward purified by a second step of affinity using Ni-NTA agarose (Qiagen, Hilden, Germany) in phosphate buffer A.

#### His_5_-SUMO-TEV-sP36 construct

*E. coli* Rosetta containing the His_5_-SUMO-TEV-sP36 construct was grown in the LB medium at 37°C under continuous shaking. At T_600_ = 0.6, the protein expression was induced by adding 100 µM IPTG (final concentration) and the cultures were incubated over night at 20°C. Cells were harvested by centrifugation at 4,000 *g* at 4°C for 20 min, suspended in the Tris-HCl buffer A (20 mM Tris-HCl, 300 mM NaCl, pH 8.0), and lysed by sonication (Gardiner, NY, USA). Afterward, the extract was centrifuged for 30 min at 13,000 *g* and 4°C. His-tagged proteins were purified by affinity chromatography in a HisTrap column (Cytiva, Marlborough, MA, USA). Proteins were eluted with the Tris-HCl buffer with 500 mM imidazole. Protein fractions were dialyzed against 20 mM Tris-HCl, 0.5 M NaCl, 2 mM DTT, pH 8.0 in the presence of TEV protease. The cleaved sP36 protein was afterward separated from undigested tagged sP36, cleaved His_5_-SUMO-TEV-tag, as well as the His-tagged TEV protease by a second step of affinity using a HisTrap column (Cytiva, Marlborough, MA, USA). Final purification was conducted using gel filtration with a S-100 Sephacryl HR column (Cytiva, Marlborough, MA, USA) and 20 mM Tris-HCl, pH 8.0, 0.15 M NaCl buffer.

#### AmtB_1_-His_6_

AmtB_1_-His_6_ was purified from heterologous overexpression in *E. coli* C43 using a solubilized membrane fraction. Therefore, cultures were grown in the LB medium at 37°C. At T_600_ = 0.6, the protein expression was induced by adding 500 µM IPTG (final concentration) and the cultures were incubated for 3 hours at 37°C. Cells were harvested by centrifugation at 6,000 *g* for 20 min at 4°C. Next, a 4 g pellet was resuspended in 4 mL of 50 mM Tris buffer (pH 7.6) and lysed by passing through the French pressure cell two times at 40 N(mm^2^)^−1^. The extract was then centrifuged again at 8,000 *g* for 20 min at 4°C to remove the remaining unlysed cells and cell debris. The cleared supernatant was transferred into new tubes and centrifuged in an ultracentrifuge (Optima XPN-100 Ultracentrifuge, Beckman Coulter, Brea, California, USA) for 1 h at 210,000 *g* and 4°C. The membrane pellet was washed with 15 mL of 50 mM Tris buffer (pH 7.6) and again centrifuged in the ultracentrifuge for 1 h at 4°C and 210,000 *g*. Afterward, the membrane proteins in the pellet were solubilized in 1 mL of phosphate buffer B (50 mM phosphate, 150 mM NaCl, 2% DDM, pH 8.0).

The solubilized membrane fraction was added on an affinity chromatography Co-NTA agarose gravity flow column (bed volume: 0.5 mL). For washing and elution steps, phosphate buffer C (50 mM phosphate, 150 mM NaCl, 0.05% DDM, pH 8.0) was used. His-tagged proteins were eluted in 0.5 mL steps using phosphate buffer C with 100 mM, 250 mM, and 500 mM imidazole.

#### MST

Proteins were purified to apparent homogeneity by affinity chromatography using Ni-NTA agarose and labeled with the RED-NHS, 2nd generation, 650 nm fluorescent dye using the Monolith NT RED-NHS lysine labeling kit according to manufacturer’s protocol (NanoTemper, Munich, Germany). RED-labeled untagged sP36 at 100 nM and His6-GlnK1 at 16 different concentrations ranging from 7.5 µM to 0.23 nM or 10 nM of sP36-RED and AmtB_1_-His_6_ in 16 dilutions ranging from 12.6 µM to 0.38 nM (all concentrations based on monomeric molecular mass) were used. The protein interaction was measured in standard capillaries (NanoTemper), 100% excitation power, and medium or high MST power (IR laser intensity). Both interactions were tested in three biological replicates. For measuring the interaction between AmtB_1_ and GlnK_1_ in dependence of sP36, RED-labeled His_6_-AmtB_1_ at 20 nM and GlnK_1_ at 16 different concentrations ranging from 480 µM to 14.6 nM and untagged purified sP36 at 480 µM or 0 µM (concentrations based on the monomeric molecular weight) were used. The protein interaction was measured in standard capillaries (NanoTemper), 20% excitation power, and medium MST power (IR laser intensity). Interactions were tested in three biological replicates.

### ITC

Standard ITC experiments were performed using an Auto-iTC200 system (MicroCal, Malvern Panalytical, Malvern, UK). Briefly, 20 µM GlnK1 was titrated with 300 µM sP36 in a buffer of 100 mM potassium phosphate, 2 mM EDTA, pH 7.0 at 25°C. Control experiments were performed by injecting the sP36 protein into the buffer. The heats of dilution were negligible. The resulting heats were integrated and normalized by ligand injected and fitted with a model for a single ligand binding site implemented in the software package Origin 7.0 (OriginLab Corporation, Northampton, MA, USA) employing user-defined fitting routines.

### SEC

SEC was conducted with 0.3 mg of His_6_-sP36, purified from homologous expression in *M. mazei,* and 0.5 mg of untagged sP36 (derived from His_6_-SUMO-sP36) using 50 mM phosphate buffer containing 300 mM NaCl and the analytical gel filtration column ENrich SEC 650 (BioRad, Hercules, USA). The protein was eluted at a flow rate of 1 mL min^−1^ with 50 mM phosphate buffer (150 mM NaCl, pH 8.0). Elutions were collected in 1 mL fractions. In order to calibrate the chromatograph, a protein mix (BioRad size-exclusion standard; #151–1901, BioRad, Hercules, USA) was used as a standard.

## SUPPLEMENTAL MATERIAL

Fig. S1 spectrum.02811-23-s0001.tif Southern blot of *M. mazei* ∆sp36 genomic DNA: to test the success of the sp36 deletion in *M. mazei.*

Fig. S2 spectrum.02811-23-s0002.tif Confidence of the predicted structure of sP36.

Fig. S3 spectrum.02811-23-s0003.tif Color-coded representation of the electrostatic potential of the surface of AmtB1 and sP36.

Table S1 spectrum.02811-23-s0004.docx Strains and plasmids used.

Table S2 spectrum.02811-23-s0005.docx Oligonucleotides used.

ASM does not own the copyrights to Supplemental Material that may be linked to, or accessed through, an article. The authors have granted ASM a non-exclusive, world-wide license to publish the Supplemental Material files. Please contact the corresponding author directly for reuse.
