# Supplementary figures and images for: Small protein mediates inhibition of ammonium transport in Methanosarcina mazei—an ancient mechanism?
Source: Microbiol Spectr. 2023 Nov 1;11(6):e02811-23. doi: 10.1128/spectrum.02811-23 (PMC10714827; doi:10.1128/spectrum.02811-23)

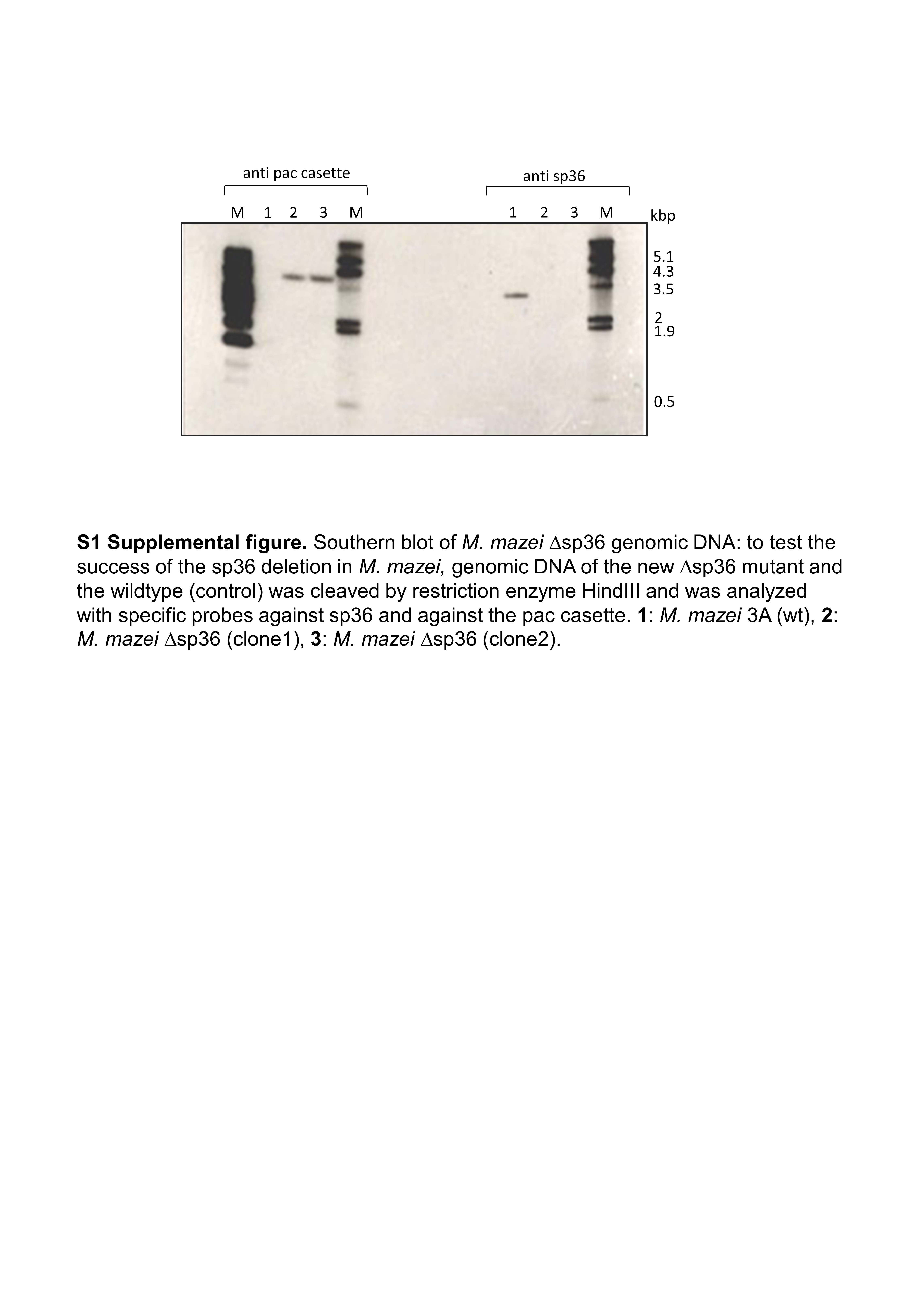

Supplement: Fig. S1 — Southern blot of M. mazei ∆sp36 genomic DNA: to test the success of the sp36 deletion in M. mazei. [file spectrum.02811-23-s0001.tif]

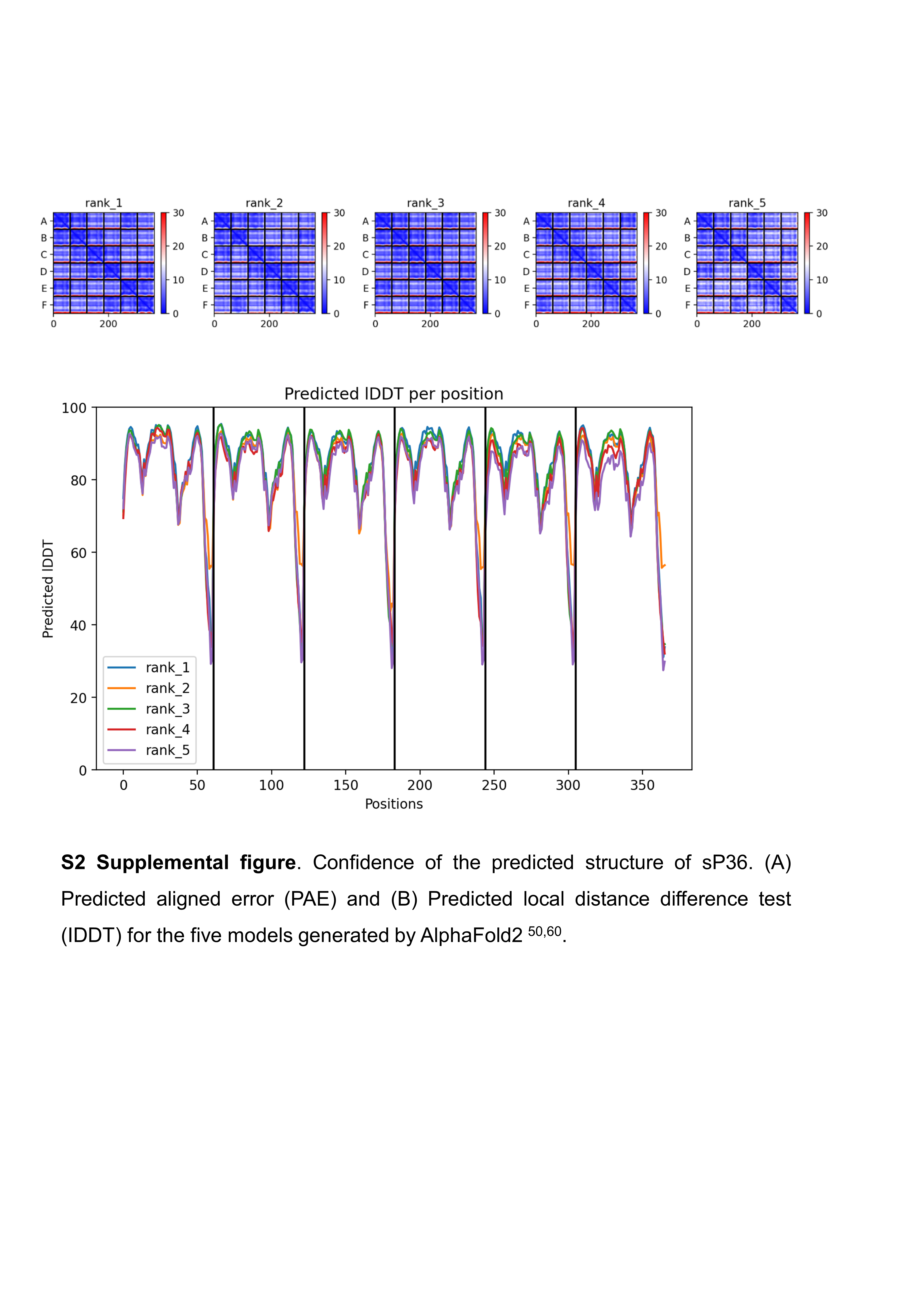

Supplement: Fig. S2 — Confidence of the predicted structure of sP36. [file spectrum.02811-23-s0002.tif]

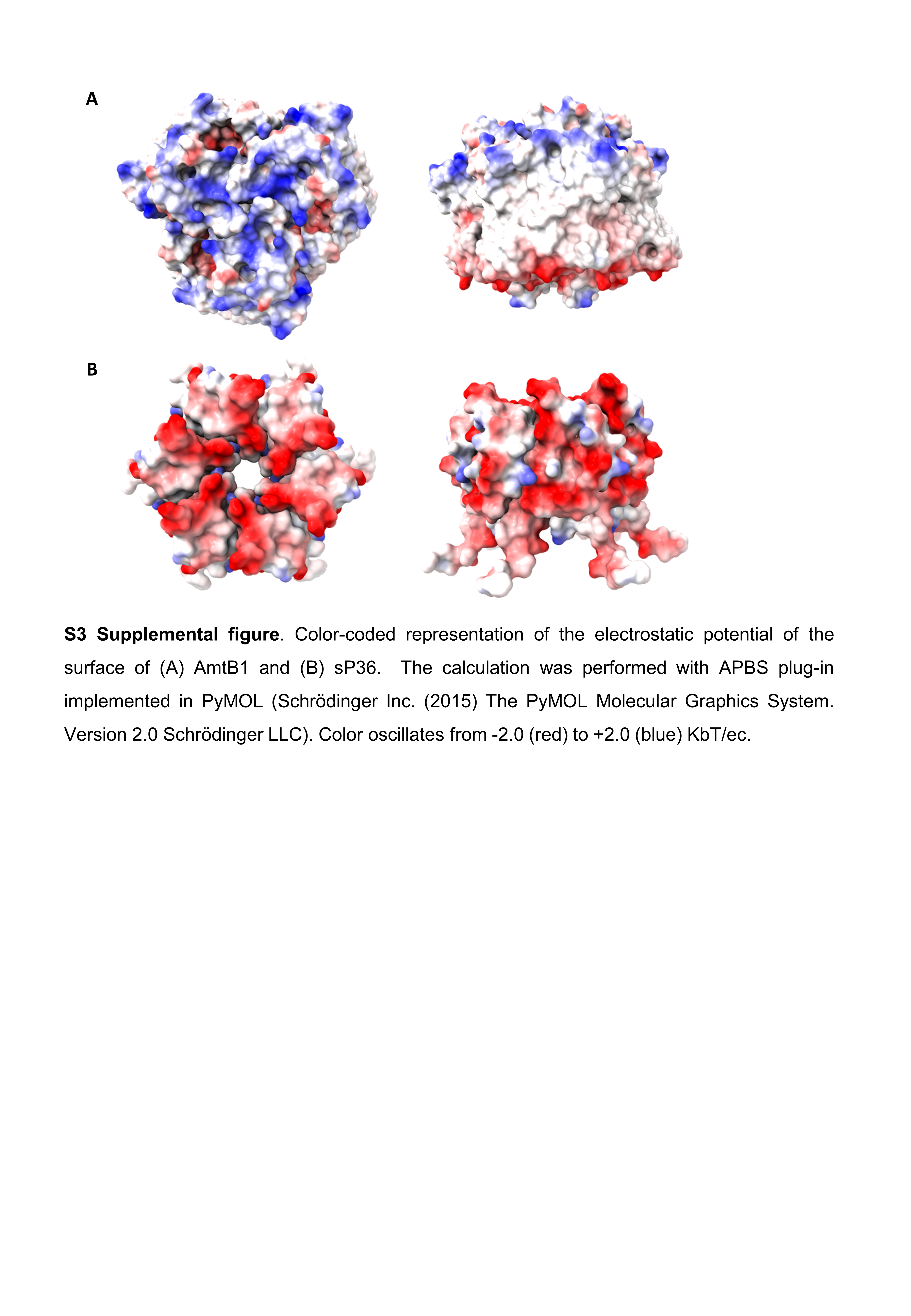

Supplement: Fig. S3 — Color-coded representation of the electrostatic potential of the surface of AmtB1 and sP36. [file spectrum.02811-23-s0003.tif]
