# Supplementary material for: Small protein mediates inhibition of ammonium transport in Methanosarcina mazei—an ancient mechanism?
Source: Microbiol Spectr. 2023 Nov 1;11(6):e02811-23. doi: 10.1128/spectrum.02811-23 (PMC10714827; doi:10.1128/spectrum.02811-23)
Supplement: Table S1 — Strains and plasmids used. [file spectrum.02811-23-s0004.docx]

## **S1 Table: Used Strains and plasmids**

| **Archaea** | | |
| --- | --- | --- |
| ***Methanosarcina mazei* Goe1** | Wildtype | German Collection of Microorganisms and Cell Cultures GmbH (DSMZ) number 3647 |
| ***Methanosarcina mazei* wt (3A)** | Wildtype,  improved growth on solid media | ^57^ |
| ***Methanosarcina mazei* ΔsP36** | *M. mazei* 3A/∆sP36 genomic deletion | This work |
| ***M. mazei* 3A pWM321/MMsORF36.N** | pRS1227 in *M. mazei* 3A Pur^R^ | This work |
| **Bacteria** | | |
| ***E.* coli DH5α** | host strain for cloning of plasmid constructs (F­-, *lacZDM15*, *recA1*, *hsdR17*, *supE44*, *lacZYA*, *argF*) | ^61^ |
| ***E.* coli BL21 (DE3) contianing pRIL** | strain for overexpression of archaeal proteins F-, *ompT hsdS(rB - mB), dcm+, Tetr gal λ (DE3), endA Hte* [*argU proL Camr*] [*argU ileYleuW Strep*/*Specr*] | Stratagene, La Jolla, USA |
| ***E. coli* C43 (DE3)** |  | ^62^ |
| **E2407** | pWM321 in *E. coli* JM109 λ pir Amp^R^, Pur^R^ | ^57^ |
| **E1570** | pET28a(+) in *E. coli* DH5α Kan^R^ | Novagen |
| **K4057** | pRS1214 in *E. coli* DH5α Amp^R^ | This work |
| **K4071** | pRS1223 in *E. coli* DH5α Amp^R^, Kan^R^ | This work |
| **K4076** | pRS1225 in *E. coli* DH5α Kan^R^ | This work |
| **K4078** | pRS1227 in *E. coli* JM109 λ pir Amp^R^, Pur^R^ | This work |
| **K4098** | pRS1240 in *E. coli* DH5α Kan^R^ | This work |
| **K4099** | pRS1240 in *E. coli* BL21 Kan^R^ | This work |
| **Plasmids** | | |
| **pmcl210** | cloning Vector, P15Aori, *lacZ*, *cat* | ^63^ |
| **pRS207** | pac cassette in pBlueskript SK | ^57^ |
| **champion pET SUMO** | expression vector, Kan^R^ | Thermo Fischer Scientific, Waltham, USA |
| **pWM321** | shuttle Vector *M. mazei* /*E. coli* *ori*R6K: pC2A replicon, Amp^R^, Kan^R^ | ^64^ |
| **pET28a(+)** | expression vector, Kan^R^ | Novagen, Darmstadt, Germany |
| **pET21a(+)** | expression vector, Amp^R^ | Novagen, Darmstadt, Germany |
| **pCR™II-TOPO®** | Plac, LacZα-ccB, AmpR, KanR, pUC ori | Thermo Fisher Scientific, Waltham, USA |
| **pRS1223** | *pCR™II-TOPO®/MMsORF36 AmpR, KanR* | This work |
| **pRS1225** | *pET28a/MMsORF36 KanR* | This work |
| **pRS1227** | *pWM321/MMsORF36.N* Pur^R^, Amp^R^ | This work |
| **pRS1240** | *pETSUMO/MMsORF36.N* Kan^R^ | This work |
| **pRS1308** | pWM321/MMsORF36 flanking regions/pac casette | This work |
| **pRS1859** | pET21a/*MM733* Amp^R^ | This work |
