# Supplementary material for: Small protein mediates inhibition of ammonium transport in Methanosarcina mazei—an ancient mechanism?
Source: Microbiol Spectr. 2023 Nov 1;11(6):e02811-23. doi: 10.1128/spectrum.02811-23 (PMC10714827; doi:10.1128/spectrum.02811-23)
Supplement: Table S2 — Oligonucleotides used. [file spectrum.02811-23-s0005.docx]

## S2 Table: Used Oligonucleotides

| **construct** | **primer** | **Sequence** |
| --- | --- | --- |
| ΔsP36 | DSP35KpnIfor | 5’-GGTACCGCCCTGCTTG |
|  | DSP35ecoRIrev | 5’-GTCATCTCCAGTAGAATTCGG |
|  | DSP35ecoRIfor | 5’-CTCAGAATTCCTGCTGATCC |
|  | DSP35BamHIrev | 5’-CGATTTTCGATGGTGGATCC |
| His_6_-SUMO-sP36 | sORF36_3_for | 5’-GTGACCATCTGGGAATACGATG |
|  | sORF36_3_rev | 5’-AAAAAATTAGAAGGCTGCATCGACATAATC |
| His_6_-sP36 | sORF36_3_forNdeI | 5’-CATATGGTGACCATCTGGGAATACGATG |
|  | sORF36_3_forNdeI | 5’-CATATGAAAAAATTAGAAGGCTGCATCGACATAATC |
| TEV cleavage site | SP36_TEV_rv | 5’-aaataaagattctcgccACCAATCTGTTCTCTGTGAGCCTC |
|  | SP36_TEV_fw | 5’-aaataaagattctcgccGGTGTGACCATCTGGGAATACGATG |
